# Metal air pollution partnership solutions: building an academic-government-community-industry collaboration to improve air quality and health in environmental justice communities in Houston

**DOI:** 10.1186/s12940-020-00590-1

**Published:** 2020-04-05

**Authors:** Elaine Symanski, Heyreoun An Han, Loren Hopkins, Mary Ann Smith, Sheryl McCurdy, Inkyu Han, Maria Jimenez, Christine Markham, Donald Richner, Daisy James, Juan Flores

**Affiliations:** 1 Center for Precision Environmental Health and Department of Medicine, Baylor College of Medicine, Houston, TX 77030-3411 USA; 2 Southwest Center for Occupational and Environmental Health, Department of Epidemiology, Human Genetics and Environmental Sciences, 1200 Pressler, Houston, TX 77030 USA; 3 Department of Statistics, MS 138, Rice University, Houston, TX 77251 USA; 4 Department of Health Promotion and Behavioral Sciences, UTHealth School of Public Health, Houston, TX 77030 USA; 5 Bureau of Pollution Control and Prevention, Houston Health Department, 7411 Park Place Blvd, Houston, TX 77087 USA; 6 Air Alliance Houston, 2520 Caroline, Houston, TX 77004 USA

**Keywords:** Academic–government-community-industry partnership, Metal air pollution, Community advisory board, Community-based participatory research, Environmental justice, Public health action plan

## Abstract

**Background:**

From 2006 to 2011, the City of Houston received nearly 200 community complaints about air pollution coming from some metal recycling facilities. The investigation by the Houston Health Department (HHD) found that while operating within legal limits, emissions from facilities that use torch cutting, a technique generating metal aerosols, may increase health risks for neighboring residents. Choosing to use collaborative problem solving over legislative rulemaking, HHD reached out to The University of Texas Health Science Center at Houston (UTHealth) to further evaluate and develop plans to mitigate, if necessary, health risks associated with metal emissions from these facilities.

**Methods:**

Utilizing a community-based participatory research approach, we constituted a research team from academia, HHD and an air quality advocacy group and a Community Advisory Board (CAB) to draw diverse stakeholders (i.e., frustrated and concerned residents and wary facility managers acting within their legal rights) into an equitable, trusting and respectful space to work together. Next, we investigated metal air pollution and inhalation health risks of adults living near metal recyclers and ascertained community views about environmental health using key informant interviews, focus groups and surveys. Finally, working collaboratively with the CAB, we developed neighborhood-specific public health action plans to address research findings.

**Results:**

After overcoming challenges**,** the CAB evolved into an effective partnership with greater trust, goodwill, representation and power among members. Working together to translate and share health risk assessment results increased accessibility of the information. These results, coupled to community survey findings, set the groundwork for developing and implementing a stakeholder-informed action plan, which included a voluntary framework to reduce metal emissions in the scrap yard, improved lines of communication and environmental health leadership training. Tangible outcomes of enhanced capacity of our community and governmental partners included trained residents to conduct door-to-door surveys, adaptation of our field training protocol and survey by our community partner and development of a successful HHD program to engage residents to improve environmental health in their neighborhood.

**Conclusions:**

Academic-government-community-industry partnerships can reduce environmental health disparities in underserved neighborhoods near industrial facilities.

## Background

Metal recycling is a robust industry in Houston, Texas with over 100 metal recycling facilities in operation [1]. Metal emissions can be generated during outdoor operations in most scrap yards, which include gas torch cutting and mechanical cutting methods that help to downsize scrap metal for eventual consumption by end users [2]. Metal torch cutting typically is of most concern because it has the potential to generate inhalable particles containing toxic heavy metals. However, little information is available about the impact on outdoor air quality from metal emissions due to torch cutting and associated health outcomes of residents in the downwind community. More is known about exposures from metal welding and torch cutting from data obtained in the occupational arena [3, 4]. Aside from potential health risks associated with this industry, there are benefits as well. These include energy savings and conservation of resources, generation of jobs and the positive impact on trade of significant U.S. exports worldwide [5].

From 2006 to 2011, the City of Houston 311 call system received nearly 200 air quality complaints related to various metal recycling facilities from nearby residents. Although some of these complaints expressed concerns about smoke, odor and dust, they also included other concerns not associated with air quality issues such as explosions, truck traffic and noise. Houston is the only major U.S. city with no formal zoning code [6]. Because of mixed land use throughout the city, industries of all types operate near residential areas that are often minority and socioeconomically disadvantaged [7]. These areas are referred to as Environmental Justice (EJ) communities because they are disproportionately impacted with increased risks of adverse health consequences associated with exposure to multiple environmental and social stressors [8, 9].

In response to the 311 calls, the Houston Health Department (HHD) Bureau of Pollution Control and Prevention (BPCP) conducted fence line air monitoring for a suite of metals in total suspended particles (TSP) at 26 metal recycling facilities during 2010–2012 using a van equipped with air monitoring equipment (a mobile ambient air monitoring laboratory, MAAML). At some of these locations, most notably those that use torch cutting, known carcinogenic metals were detected in the ambient air (e.g., nickel compounds) [10] in addition to those that can cause non-carcinogenic adverse health effects (e.g., manganese and cobalt) [11]. Subsequently, HHD used the fence line air monitoring data in a health risk assessment and found increased cancer risks from metal air pollution at some locations even though the facilities were operating within legal limits (i.e., by permit by rule) [12]. These findings were highlighted in articles that appeared in Houston’s daily newspaper [13–15] and were later published in the peer-reviewed literature [1]. In response, a task force was created by the Recycling Council of Texas, the Institute of Scrap Recycling Industries (ISRI) and the Gulf Coast Chapter of ISRI. The Metal Recycler Task Force reached out to HHD to communicate their critique of the approach taken in collecting air measurements and in conducting the risk assessment. Concerns focused on the size fraction of particles that were collected (i.e., TSP); the method that was used to estimate upwind/background concentrations from deployments that were not specific to the monitored metal recycler locations; collecting air measurements at the fence line only and not in the neighborhoods where individuals reside; and uncertainty in the assessment of hexavalent chromium levels because they were estimated from concentrations of total chromium.

The HHD, not being a research entity, needed support to better understand the risk and, if the risk was identified, a mechanism to mitigate it. The HHD approached investigators at The University of Texas Health Science Center at Houston (UTHealth) School of Public Health who, with HHD, subsequently partnered with Rice University, Air Alliance Houston (AAH), an EJ advocacy group, neighborhood civic leaders and the Metal Recycler Task Force, to determine next steps. Stakeholder feedback aided development of the grant submission, which was designed to be inclusive of governmental, community and industry views. In 2014, as part of the National Institute of Environmental Health Sciences (NIEHS) “Partnerships for Environmental Public Health” (PEPH) network through their Research to Action program, the partnership was awarded a grant entitled, *Solutions to Metal Air Pollution in Disadvantaged Neighborhoods* (or “MAPPS” for Metal Air Pollution Partnership Solutions) (R01ES023563) to address and mitigate potential adverse health impacts in communities close to metal recycling facilities.

The primary purpose of this article is to describe the community-based participatory research (CBPR) approach [16, 17] used to build a unique collaboration among academics, HHD, AAH, community residents and metal recycling representatives in the MAPPS project. In this review, we document efforts of the partnership to address community and industry concerns and illustrate outcomes of our combined endeavors. In addition, we comment on lessons learned when engaging a diverse group of stakeholders to address collective goals to improve air quality and environmental health in neighborhoods located adjacent to metal recycling facilities in Houston.

## Methods

### Overview

**Fig. 1:**
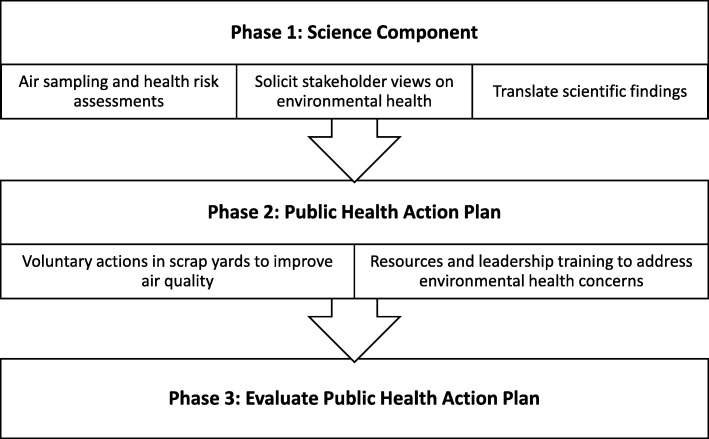
Three phases in the Metal Air Pollution Partnership Solutions (MAPPS) research to action project

MAPPS is a project comprised of three phases: Phase 1: A Science Phase, Phase 2: A Public Health Action Plan and Phase 3: An Evaluation Phase (see Fig. 1). The goals of Phase 1, the scientific phase, were three-fold. First, to assess possible increased health risks due to metals emitted from nearby metal recycling facilities, we conducted community air monitoring from September 2015 to May 2017 (*n* = 63 days) in four selected neighborhoods measuring inhalable particles with diameters that were 10 μm and smaller (PM_10_) at four sampling locations simultaneously (i.e., at an upwind location of the metal recycling facility, one at the fence line and two downwind locations) in each neighborhood. The collected samples were analyzed for 10 metals (arsenic, silver, cadmium, chromium, cobalt, manganese, iron, nickel, lead and selenium) and the measurements were used to estimate neighborhood- and location- specific cancer and non-cancer health risks associated with metal emissions from metal recycling facilities among adults who lived nearby. Second, we gathered information about stakeholder’s views and concerns about their neighborhood and environmental health using mixed methods through key informant interviews (*n* = nine), focus groups (*n* = six) and door-to-door community surveys (*n* = 375), based on language preference of the participant (English or Spanish). Third, working with the Community Advisory Board (CAB), we translated scientific findings. The other two components of the project (Phases 2 and 3) were focused on developing, implementing and evaluating a multilevel, evidence-based [18] action plan to improve environmental health in the impacted communities. Details regarding the air sampling, health risk assessment, methods for gathering stakeholder views about environmental health and the community-driven public health action plan will be reported elsewhere. Here, we describe how we applied CBPR principles and iterative engagement processes to establish a strong partnership and worked to develop a balance between research and action for the mutual benefit of all partners. All MAPPS activities were approved by UTHealth’s Institutional Review Board (IRB), the Committee for the Protection of Human Subjects.

### MAPPS communities

**Fig. 2:**
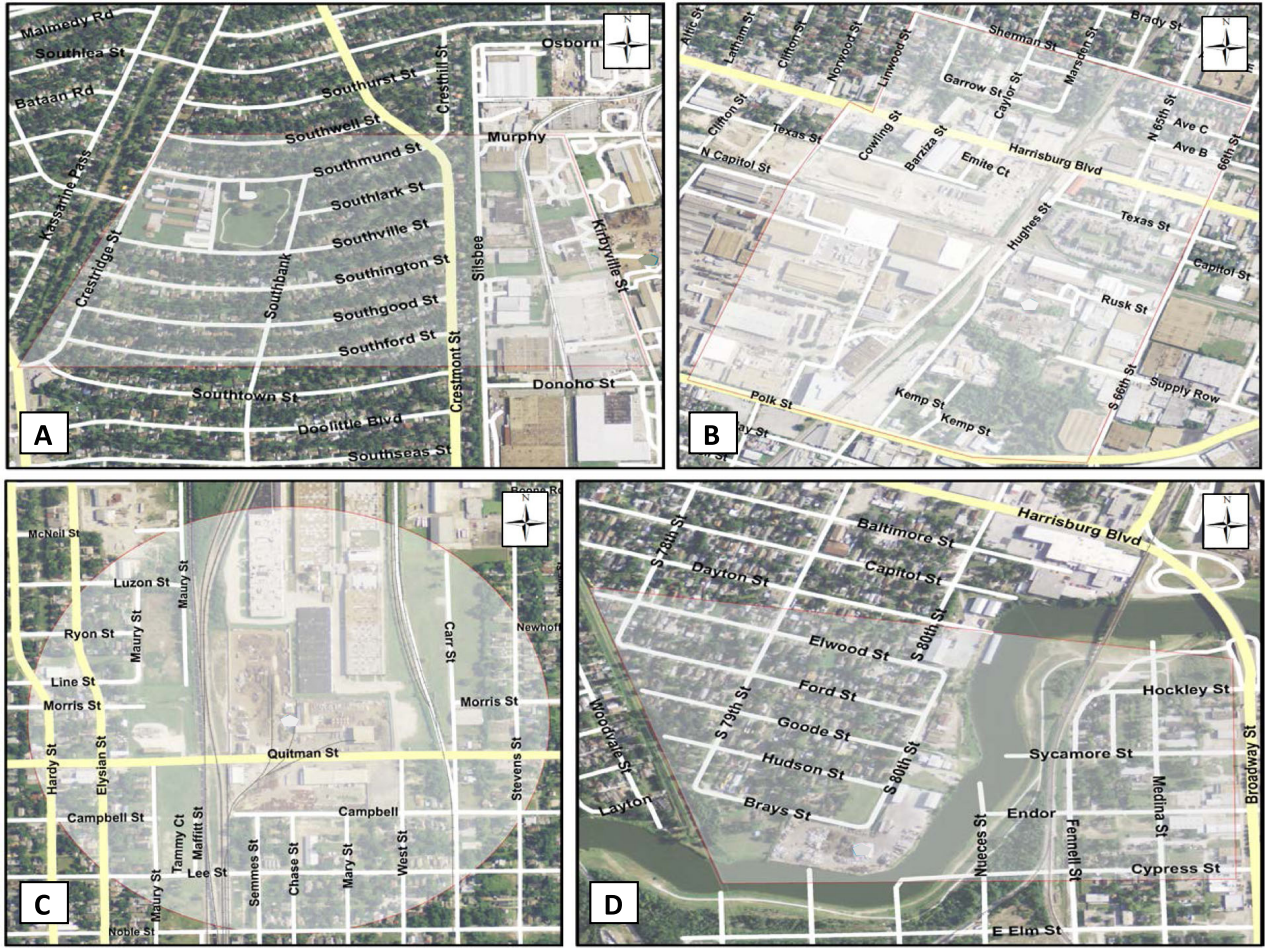
Metal Air Pollution Partnership Solutions (MAPPS) communities: (**a**) South Park, (**b**) Magnolia Park West, (**c**) Fifth Ward/ Northside and (**d**) Magnolia Park East

The MAPPS communities (four metal recyclers and adjacent residential areas) were selected based on the following considerations: 1) availability of previous air monitoring results conducted by HHD in response to 311 complaints, 2) a metal recycling facility with an outdoor operation in a neighborhood with a minimal number of other known nearby sources of metal emissions and 3) a metal recycling facility in an area with at least one residential area next to the facility in which four sampling locations could be identified within a line without physical obstructions. Four metal recycling facilities were identified in the communities of South Park (Fig. 2a), Fifth Ward/Northside (Fig. 2c), Magnolia Park East (Fig. 2d) and East Lawndale. The metal recycling facility in East Lawndale went out of business in November 2015 and was replaced with a facility operating in Magnolia Park West (Fig. 2b).

Magnolia Park is located in eastern Harris County, near the Port of Houston. Magnolia Park is one of the city’s oldest Hispanic communities, dating back to the 1930s [19]. The Fifth Ward/Northside communities are located close to downtown Houston. The Fifth Ward is one of Houston’s original six wards (the political and geographic areas that were established when Houston was founded and incorporated in the 1830s) and it became home to freedmen after the Civil War [20]. Near Northside [21], located north and west of Fifth Ward, consists of homes that surround commercial properties and it is predominantly Hispanic. South Park [22] is an African-American community, originally developed during the 1950s, which is located in the south-central area of Houston. It is directly south of Interstate 610, which forms a 38-mile loop around the city. Table 1 provides a snapshot of the sociodemographic profile of the populations within a 0.25-mile buffer of the four metal recycling facilities selected in the study.

**Table 1:** Sociodemographic profile of residents living within a 0.25 mile of four metal recycling facilities in the Metal Air Pollution Partnership Solutions (MAPPS) study, Houston, Texas [23]

Characteristic	Magnolia Park East	Magnolia Park West	Fifth Ward/ Northside	South Park	USA average
% Minority	99 (mostly Hispanic)	92 (mostly Hispanic)	92 (mostly Hispanic)	100 (mostly Black)	37
% Low income^a^	60	67	67	66	35
% Linguistically isolated^b^	42	52	20	5	5
% < High school education	51	41	51	39	14

^a^ Low-income is defined as the percentage of a block group’s population in households where the household income is less than or equal to twice the federal poverty level

^b^ Linguistic isolation is percentage of people in households in which all members over age 14 years speak English less than “very well”

### Academic-government-community research team

The pre-grant academic-government-community group morphed into a formal research team comprised of academic (UTHealth School of Public Health and Rice University), governmental (HHD) and community (AAH) partners. University members of the research team represented multidisciplinary training and experience in behavioral sciences, CBPR, environmental chemistry, environmental epidemiology, exposure science, health promotion, intervention mapping [18] and risk assessment and evolved over time to include additional expertise in toxicology, social sciences and qualitative research methods. A bi-lingual Hispanic community organizer with 40 years of experience working on social justice issues among Latinx brought unique expertise to the research team, including participation on prior CBPR projects [24, 25], and served a critical role as a bridge between academic and community partners [26, 27]. The research team held bi-weekly or monthly meetings at AAH, HHD and UTHealth to guide all research activities.

### Expanded partnership with residents and industry representatives

During the first year of the project, a CAB was formed to engage primary stakeholders from the affected communities as “MAPPS Partners”. AAH recruited resident leaders in Magnolia Park, Near Northside, Fifth Ward or South Park who lived or grew up in and/or had strong ties to their communities (e.g., as a civic club president, a religious leader or a member of the Board of Trustees for Houston Community College). The Metal Recycler Task Force assisted in identifying CAB representatives from the participating facilities as well as their attorney. In addition, four “at-large” members from the community and the metal recycling industry were identified to represent broader perspectives. Eighteen members were selected to serve on the CAB including six residents, six metal recyclers and six research team members and the membership has grown to 29 active members including seven residents, nine metal recyclers and 13 research team members.

Throughout the project, the CAB generally met once a month at different locations including venues within study neighborhoods. We offered rides and served a light lunch to encourage participation. CAB meetings were often facilitated by the principal investigator (P.I.) (E.S.) and structured around discussions on updates on ongoing activities, study materials, research findings and next steps along with meeting evaluations at the end of each session. Mini-trainings and small-group breakout sessions were used to facilitate input as well. CAB meetings were open to anyone wishing to attend. However, when it was time to discuss risk assessment and community survey results, we held closed meetings to allow time to interpret the results and develop a public health action plan. Prior to holding closed meetings, all members signed an agreement to keep discussions confidential. Additionally, we worked together to prepare neighborhood-specific reports written in a manner accessible to the lay public and organize community forums to disseminate the findings.

To encourage greater participation and input from residents, as well as to address the knowledge gap between residents and recyclers, we scheduled resident CAB luncheon meetings a few days before CAB meetings. The luncheon meetings provided not only an opportunity to socialize and bond as a group but allowed resident members opportunities to acquire scientific knowledge, be informed of upcoming meeting agenda items and provide input or raise questions in a less formal and small group setting. Besides the luncheons, our community organizers followed up on an ad-hoc basis with resident CAB members to gain their impressions of CAB meetings and ask about questions and concerns that might not have been addressed.

## Results

### Diversity among the CAB

We constituted a CAB of diverse key stakeholders across the study area. Building on strengths and resources within the community, residents provided a deep historical and cultural understanding of the neighborhoods and the recyclers brought knowledge about metal recycling industry operations. Our community partner, AAH, brought expertise in environmental advocacy and community outreach and our governmental partner, HHD, provided resources and expertise in air monitoring, air pollution mitigation measures and outreach to residents who expressed environmental health concerns through the city’s 311 call system. Importantly, HHD agreed not to issue enforcement violations to MAPPS metal recycling partners during the project period and instead, if necessary, to work with metal recyclers for timely resolution for corrective actions.

### Specifying CAB roles and responsibilities and a MAPPS communication plan

Early on, the CAB worked on process-related items, which included shortening the project name and developing operating norms articulated in a Memorandum of Roles and Responsibilities of Partners and a Communication Plan. The Memorandum specified the goals of the project and lead roles or responsibilities of partners. Common to all partners was the expectation to “regularly share information and provide feedback, guidance and support to all partners”. The Communication Plan guided outreach activities and messages. Focusing on different activities, the CAB defined the following: “What are our messages?”; “Who is our target audience?”; “What tools will be used to deliver our messages?”; “How will we communicate our messages?”; “Who will deliver the messages?”; and “What is our timeline?”.

The CAB proposed ways to make MAPPS more visible in our target communities and inform residents about the project. We attended community meetings and distributed a project brochure and flyers announcing community air monitoring and inviting residents to visit while sampling was being conducted. Also, the MAPPS metal recyclers provided a “direct-communication” line to address resident’s concerns and the HHD made changes in Houston’s’ 311 call system enabling a 24-hr response to investigate environmental health concerns associated with metal recycling operations (both of which represent elements of the project’s public health action plan that emerged during the formative (scientific) phase of the project). All materials were neighborhood-specific and prepared in English and Spanish. There was a consensus among the CAB that developing a bi-lingual webpage for the project would serve as an appropriate vehicle to provide project updates and aid in outreach efforts (https://go.uth.edu/MAPPS).

### CAB role in phase 1: science component

During the science phase of the study, the roles and responsibilities of the CAB evolved from “functioning participation” to “iterative participation” through which they became more involved in decisions [28]. One of the first tasks was to evaluate the sampling design and methods for air monitoring in communities that were described in a Standard Operating Procedures (SOP) manual, which was followed by providing input on key informant interview questions and focus group probes as well as on questions and the Manual of Operating Procedures (MOP) for the community surveys. CAB members were instrumental in recruiting key informant and focus group participants, many of whom lived in our MAPPS neighborhoods or worked at the study’s metal recycling facilities. Likewise, the CAB helped to identify residents as potential interviewers for the community survey and both CAB resident and metal recycling members were involved in the hiring process. In addition, the CAB provided cultural insight with suggestions on how and when to administer the survey to yield greater participation and which incentives to provide study participants, as well as a need for a bilingual glossary. A subcommittee of bilingual resident CAB members reviewed translated (Spanish) materials and evaluated the bilingual fluency of potential field staff during the interview process. The human subjects research and interviewer training materials that we developed for field staff were shared with all partners and the training was made available to our community partner (AAH) and the CAB to increase their environmental health literacy and research capacity.

### CAB role in phase 2: public health action plan

Over the course of several months, trust and capacity grew during closed CAB meetings, mini-trainings and sub-group meetings among residents and metal recyclers who worked together to interpret findings from the risk assessment and community surveys. In developing the action plan, the CAB metal recyclers took the initiative on developing a framework for making voluntary changes in the scrap yards to improve air quality and for improving communication with residents, while resident CAB members led activities to gain input from their neighbors on the action plan and plan the community forums. Together, we made decisions on what information was to be included in neighborhood-specific community reports and when and where to disseminate the findings and elements of the action plan.

### Translating CBPR principles into practice

**Fig. 3:**
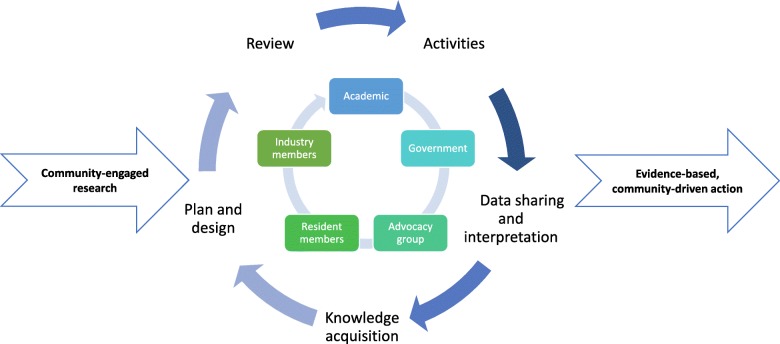
Cyclical engagement framework of the Metal Air Pollution Partnership Solutions (MAPPS) project to build collaborative and equal relationships among partners

Table 2 describes selected outcomes based on MAPPS activities that were built around underlying CBPR principles [29, 30]. As the project unfolded, several challenges emerged: 1) imbalance in knowledge, power and resources among partners, 2) difficulty in maintaining connections and communications among all partners, 3) lack of understanding, by some CAB members, of the need to adhere to the IRB protocol, 4) translation of scientific results into accessible language and 5) frustrations with the time delay between research activities and development of the action plan. To address these challenges, we applied a cyclical engagement framework ensuring collaboration and worked to ensure an equitable decision-making process for all project phases (see Fig. 3). This iterative process was used to inform, educate and empower the partners, as well as to cultivate trust and facilitate partnerships that matured with time.

**Table 2:** CBPR principles and MAPPS project outcomes

CBPR Principles [26, 29]	MAPPS Outcomes
Recognize community as a unit of identity	Partnership among residents and metal recyclers in four neighborhoods who worked together to address an environmental health concern
Build on strengths and resources within the community	Shared expertise and learning among partners
Facilitate collaborative, equitable partnership in phases of the research	Articulated partner roles and responsibilities; Facilitated Community Advisory Board (CAB) meetings to allow for and encourage equal participation; Increased level of CAB engagement by relationship- and trust-building
Promote co-learning and capacity building among all partners	Improved knowledge about environmental health, neighborhoods and metal recycling through co-learning opportunities at meetings, workshops, tours and training activities; Adaption of MAPPS community survey protocol for other activities
Integrate and achieve a balance between research and action for the mutual benefit of all partners	A multi-faceted community-driven and evidence-based action plan based on research findings; Development of HHD Block Captain Program
Emphasize local relevance of public health problems and ecological perspectives that recognize and attend to the multiple determinants of health and disease	Lay reports of research findings that communicate the history of the project and major findings, as well as the impacts of the broader biological, environmental and social contexts on health and well-being
Involve systems development through a cyclical and iterative process	A decision-making process facilitated by regularly scheduled meetings that encouraged (sometimes diverse) input from all partners; Changes in process based on regular evaluations of CAB meetings
Disseminate findings and knowledge gained to all partners and involving all partners in the dissemination process	CAB-driven translation and dissemination of research findings at community forums and in scientific and lay reports
Establish a long-term commitment to the process	Sustained CAB commitment and involvement through having ground rules for the decision-making process; facilitated and respectful discussions to resolve differences; Team and tool building exercises to promote trust and develop relationships

The CAB meetings created “invited spaces” [31] for members to build capacity and empower themselves. Evaluation of CAB meetings allowed for a systematic means to gain input on both meeting processes, as well as content. Recurring feedback included comments about the appreciation of the role of the facilitator and the transparent process and the safe environment that was provided to promote open discussion, along with suggestions to increase community residents’ participation. Suggestions for future agenda items and training were also sought to allow the CAB to identify areas they felt required additional review or input. For example, in discussing air monitoring and risk assessment results, resident members asked for less technical summaries, whereas the metal recyclers requested additional details. In response, we scheduled subgroup meetings with a couple of the metal recyclers to answer their questions who, in turn, began to take greater responsibility in representing the views of the metal recyclers on the CAB (a role that had been previously assumed by the Metal Recyclers Task Force leader and the attorney representing the metal recyclers). We also held small-group or one-on-one sessions with resident CAB members to communicate scientific findings using more accessible language. Later on, resident CAB members volunteered to host “house meetings” to inform the action plan and promote the MAPPS community forums in their neighborhoods. This transition is an example of how the process of inclusion and information sharing led to greater participation and empowerment.

## Discussion

The genesis of the MAPPS project was rooted in residents’ concerns, communicated through Houston’s 311 call system, about smoke, odors and dust emanating from nearby metal recycling facilities, and the resultant air quality investigation conducted by the HHD BPCP. Subsequent coverage about the monitoring results in the city’s local newspaper [13–15] served to heighten community concerns about the environmental impact of metal recycling facilities located in their neighborhoods. The lack of zoning within Houston raised an EJ issue, as many of these facilities and other industries are in predominantly poor and minority neighborhoods [7]. Building on questions raised by Houstonians and the initial air monitoring conducted by HHD, we developed a research plan, based upon the CBPR model, to address resident concerns. We conducted community air monitoring to measure metals in the air emitted primarily from metal recycling facilities, performed health risk assessments and gathered information about stakeholder perceptions on environmental health as well as on ways to improve communication. Central to the MAPPS project are the intervention and evaluation phases to develop, implement and assess a public health action plan based on risk assessment and community survey findings. Hence, the research to action components of the project align with the CBPR approach to actively engage those impacted by the issue being studied for the purpose of arriving at meaningful and sustainable solutions to eliminate health disparities [26].

Applying CBPR principles was key to the research and action components of our project. One of the underlying principles of CBPR is to recognize community as a unit of identity [26, 32], i.e., to work with individuals who identify with a larger group because of membership and engagement in a faith-based organization, a neighborhood, a political group or a non-governmental organization. Many CBPR partnerships include community groups and governmental entities [33], as does ours. However, the MAPPS project expanded upon traditional partnerships to include representatives from the impacted neighborhoods and from industry. This approach allowed industry members and residents of the project to work together side-by-side with a view of each other as allies, as opposed to adversaries, as we sought sustainable solutions to collectively address community concerns. Also, the project provided an opportunity for the HHD BPCP to partner and work together to resolve issues in ways that had not been attempted before and to consider industry as part of the community. This expanded view of the importance of communication and partnership evolved among all partners.

Another accepted principle of the CBPR process is that of equal partnership and shared responsibility in all phases of the research process [2]. Community (AAH) and governmental (HHD) partners shared budget resources from the grant, but the PI assumed overall control over the project and assumed responsibility for oversight and integration of all aspects of the project, including those spearheaded by the sub-contracting organizations. The research team met regularly and worked together to coordinate plans, communications and outreach throughout all phases of the project. Obtaining written feedback on drafts of materials was more difficult than receiving input orally during regularly scheduled meetings. We encouraged full participation from all members at meetings to develop, implement and monitor project activities. However, consistent with previous reports [34], feedback and involvement among community members sometimes waned when discussions became more technical; when there were frustrations with the academic approach that required adhering to IRB protocols or documenting activities (e.g., interactions with residents or civic and church leaders that would provide evidence of expanded community engagement for evaluation purposes); or when there were other demands on their time. This sometimes resulted in academic researchers assuming a greater role in activities, which served to shift the balance of power among partners. Nonetheless, the academic members of the team made efforts to stay aware of these resulting power imbalances and address them to the extent possible.

CBPR is challenging because of the substantial investment in time that is required to engage community partners [35, 36]. In our study, engagement took place at multiple levels, involving interactions between academics, government and community groups as research partners, as well as interactions between research partners and CAB members, i.e., residents and metal recyclers. Laying the groundwork for the partnership involved spending time on process-related activities to define the partnership and determine how the CAB would operate, as well as to respond positively to invitations to activities and events unrelated to the MAPPS project. The team approach required adaptation and flexibility as progress in achieving project goals was considerably slowed to ensure adequate time for engagement and buy-in of all partners. For example, more meetings than originally planned were needed to allow for full review and approval by the CAB of the project’s brochure (three meetings); key informant interview guides (six meetings); community surveys (five meetings); risk assessment (technical) reports (four meetings); and community reports (four meetings).

CAB members expressed frustration with the amount of time that it took to carry out specific research activities. In hindsight, it might have been helpful to have more clearly explained to CAB members the nature of the research process at the beginning as well as during different stages of the project to help them understand the iterative nature of research. There was also a lack of understanding of the need to adhere to an IRB protocol. For example, there were two protocol deviations due to a Facebook post that highlighted remuneration for participating in our community survey and a webpage post of a picture of a resident completing the survey. In both instances, steps were taken to remove posts as soon as they were discovered and explain why such posts did not provide adequate human subjects assurances. The protocol deviations were reported to our IRB with plans to address them moving forward.

We recognized a power-imbalance between our two diverse groups of stakeholders serving on the CAB. First, the knowledge base about areas pertinent to the project (e.g., metals; metal recycling operations; regulatory requirements of the industry; scientific terminology; environmental pollution and health risk) differed between metal recyclers and residents. Second, the metal recyclers had access to resources that were not available to residents. As examples, the Metal Recyclers Task Force solicited services of an attorney (who has been a member of the CAB from the start of the project), as well as external consultants who provided review of the grant application, the SOP for air monitoring and the risk assessment reports. To address this imbalance, the research team hired an outside consultant to represent community members regarding the interpretation of results from the risk assessment. This power imbalance was also countered in part by AAH, an EJ advocacy group, being a grantee on the study. Finally, the professionalism, energy and commitment of CAB members led to greater trust with each other and this served to diminish the power imbalance as well.

We also recognized an inherent difference in our CAB membership in that the metal recyclers participated during their normal workhours whereas resident members served as volunteers who had to take time from their daily routines or work in order to participate. While we provided lunch prior to our CAB meetings to show appreciation of resident members’ time and provided gift cards a few times throughout the project, it would have preferable to have budgeted annual stipends for them. Another challenge was differing priorities in that the project was of central interest to metal recyclers, whereas residents had broader environmental health concerns that extended beyond the scope of the MAPPS study. The research team did its best to provide resources that could help address resident concerns for matters tangential to the project (e.g., one resident was provided information about HHD resources on lead abatement due to concerns about the potential health impact of lead in paint inside her home).

Mutual learning and capacity building underpin the CBPR process [26]. MAPPS provided opportunities for academic researchers, HHD and AAH to view a single environmental health issue through the lens of a researcher, an advocate, a regulator, resident or metal recycler. The importance of education is central to capacity building [27] and we undertook several activities to heighten understanding about the project: a tour of a metal recycling facility (coordinated by the head of the Metal Recyclers Task Force that was preceded by a hosted lunch); a tour of the MAAML; mini-workshops on air sampling, risk assessment and community surveys; and an invited presentation given by the Texas Commission on Environmental Quality (TCEQ) on the regulatory framework in the permitting process. In CAB meetings, we noted resident CAB members deferred to researchers regarding some of the more technical aspects of the project (e.g., community air monitoring or risk assessment methods). While we attempted to communicate in lay language, the inadvertent use of scientific jargon may have caused frustration and limited full participation of CAB members, as has been noted by others [27].

We adopted and used a consensus decision-making approach throughout the project. Research team meetings and CAB meetings were structured to allow for transparent communication and open dialogue. Often, we organized individual or small group meetings with CAB resident and metal recycling members to review study materials for upcoming CAB meetings to facilitate the discussion and decision-making process. This approach allowed everyone the opportunity to examine the issues and discuss their perspectives, share information and participate in the process. However, engagement was sometimes limited when we attempted to make decisions that built on our research findings. For example, the concept of risk was difficult to grasp, and this led to the research team being asked to determine whether a risk level was “safe” or not.

The CAB generally reached consensus, but not always. For example, the metal recyclers repeatedly asked for air sampling results as they were being collected, but the study protocol had been designed to share these results after all monitoring was completed. There were also differences in opinion among the outside consultants (who were hired by the metal recyclers and the project) about interpretation of risk assessment results and among CAB members about whether these risks should be compared to risks from individual lifestyle choices like smoking. There was both support for, and opposition to, suggestions to include policy initiatives to regulate the metal recycling industry as part of the public health action plan. In the end, this was tabled in part because of insufficient time on the grant to enact legislative or regulatory changes and was to be considered later as part of longer-range initiatives.

Our industry and resident partners played active roles in developing and implementing our public health action plan. Voluntary actions on the part of our recycling industry partners to change practices, processes or conditions in the scrap yard to minimize emissions from metal recycling facilities and improve communication with residents were key elements of the action plan. Without these voluntary actions, the timeline for risk mitigation would have been extensively delayed. Resident CAB members also participated in meaningful ways by developing a colloquial version of key messages from our research findings and organizing and holding “house meetings” to solicit broader input from residents on the public health action plan. Several members of the CAB (from both our resident and metal recycling groups) participated in our Environmental Health Leadership Training. Moreover, our activities led to outcomes broader than the project. For example, AAH hired one of the field interviewers trained for our survey and adapted our survey protocols for use in another community. In addition, HHD developed a program (in response to focus group findings) funded by the de Beaumont foundation’s BUILD program [37] to train volunteers in Near Northside as “block captains” who serve as points-of-contact for residents to communicate their environmental health concerns to the HHD and for HHD to promote their environmental health programs (e.g., childhood lead surveillance program, lead abatement program and asthma prevention and control program).

## Conclusions

The unique partnership and CBPR approach described in this paper show how a positive model can be used to build partnerships across different sectors to address environmental health concerns in underserved communities near industrial areas. Engaging a diverse group of partners, including residents and industry members, was challenging. However, a multi-year effort that built on open discussions and transparency fostered trust that led to a multi-pronged action plan. Our experience provides evidence of the importance of community and industry involvement, in addition to academia, government and advocacy groups, to create a shared vision among partners that will heighten success in achieving sustainable outcomes that improve environmental health in EJ neighborhoods.

## Data Availability

Not applicable.

## Abbreviations

AAH: Air Alliance Houston
BPCP: Bureau of Pollution Control & Prevention
CAB: Community Advisory Board
CBPR: Community-Based Participatory Research
EJ: Environmental Justice
HHD: Houston Health Department
IRB: Institutional Review Board
ISRI: Institute of Scrap Recycling Industries
MAAML: Mobile Ambient Air Monitoring Laboratory
MAPPS: Metal Air Pollution Partnership Solutions
MOP: Manual of Operating Procedures
NIEHS: National Institute of Environmental Health Sciences
PEHP: Partnerships for Environmental Public Health
PI: Principal Investigator
SOP: Standard Operating Procedures
TSP: Total Suspended Particles
UTHealth: The University of Texas Health Science Center at Houston
